# Preclinical Models of Cardiac Disease: A Comprehensive Overview for Clinical Scientists

**DOI:** 10.1007/s13239-023-00707-w

**Published:** 2024-01-16

**Authors:** Elisa C. H. van Doorn, Jorik H. Amesz, Amir H. Sadeghi, Natasja M. S. de Groot, Olivier C. Manintveld, Yannick J. H. J. Taverne

**Affiliations:** 1 Translational Cardiothoracic Surgery Research Lab, Department of Cardiothoracic Surgery, Erasmus Medical Center, Rotterdam, The Netherlands; 2 Translational Electrophysiology Laboratory, Department of Cardiology, Erasmus Medical Center, Rotterdam, The Netherlands; 3 Department of Cardiology, Erasmus Medical Center, Rotterdam, The Netherlands

**Keywords:** Translational research, Cardiac disease, Preclinical modeling, In vitro, Ex vivo, In vivo, In silico

## Abstract

For recent decades, cardiac diseases have been the leading cause of death and morbidity worldwide. Despite significant achievements in their management, profound understanding of disease progression is limited. The lack of biologically relevant and robust preclinical disease models that truly grasp the molecular underpinnings of cardiac disease and its pathophysiology attributes to this stagnation, as well as the insufficiency of platforms that effectively explore novel therapeutic avenues. The area of fundamental and translational cardiac research has therefore gained wide interest of scientists in the clinical field, while the landscape has rapidly evolved towards an elaborate array of research modalities, characterized by diverse and distinctive traits. As a consequence, current literature lacks an intelligible and complete overview aimed at clinical scientists that focuses on selecting the optimal platform for translational research questions. In this review, we present an elaborate overview of current in vitro, ex vivo, in vivo and in silico platforms that model cardiac health and disease, delineating their main benefits and drawbacks, innovative prospects, and foremost fields of application in the scope of clinical research incentives.

## Introduction

Cardiac disease, including heart failure, coronary heart disease, and arrhythmias, remains the leading cause of death worldwide, taking millions of lives each year [1]. Despite significant advances in treatment and prevention over the past few decades, the incidence of cardiac disease is still rising, and mortality and morbidity are still high [2]. Over the past several years, our understanding of the cardiac system and its myriad of pathophysiologies has greatly progressed as a result of preclinical modeling yet characterized by inherent limitations of each model.

Historically, animal models have markedly contributed to the present knowledge of cardiac disease by characterizing disease mechanisms and uncovering new therapeutic targets [3]. However, animals show genetic and metabolic differences compared to humans and therefore interspecies extrapolation frequently results in significant pharmacokinetic mismatches [4]. In addition, there has been an increase in controversy and opposition to the use of animal models. This has prompted the exploration of other model types, ranging from simple single cell constructs towards multicellular and more complex in vitro platforms, alongside other modalities such as ex vivo models and in silico constructs. While ex vivo models offer a close approximation of the heart’s natural structure, they are often derived from animal sources and limited by their short-term applicability. In silico models provide a valuable platform for computer simulations and predictions, but require external input based on real-life experimentation. Yet, in vitro models often fail to fully recapitulate the intricate three-dimensional (3D) architecture of the heart, regularly rendering them unable to meticulously investigate disease mechanisms and identify key targets for drug development [5]. The limited ability of preclinical models to adequately predict the efficacy, safety, and toxicity of novel therapeutic agents is marked by a clinical trial failure rate of up to 90% [6]. Hence, there still is an ever-growing demand for preclinical platforms that accurately mimic the in vivo complexity of the heart and its vasculature.

To enable clinical translation, cardiac disease models should closely resemble human in vivo features, replicating disease phenotype and its underlying causality. Every model owns its unique set of benefits and drawbacks and not every research model is equally adequate for achieving a certain research aim [4]. Although several reviews do delineate specific sets of models, current literature lacks an elaborate and consistent synopsis of the full scope of contemporary platforms for cardiac disease. In addition, the majority of these reviews tend to focus on fundamental science and scientists with expertise in the preclinical research field.

Therefore, the aim of this review is to present a comprehensive overview of currently available preclinical cardiac disease models to clinical scientists, outlining their advantages and disadvantages, field of application, and perspectives on future advances. The guideline is accordingly intended to facilitate selection of preclinical models that optimally suit the character of respective (clinical) research queries and to stimulate clinical researchers to investigate new preclinical modalities for cardiac research.

## Cardiac Disease Models

**Fig. 1:**
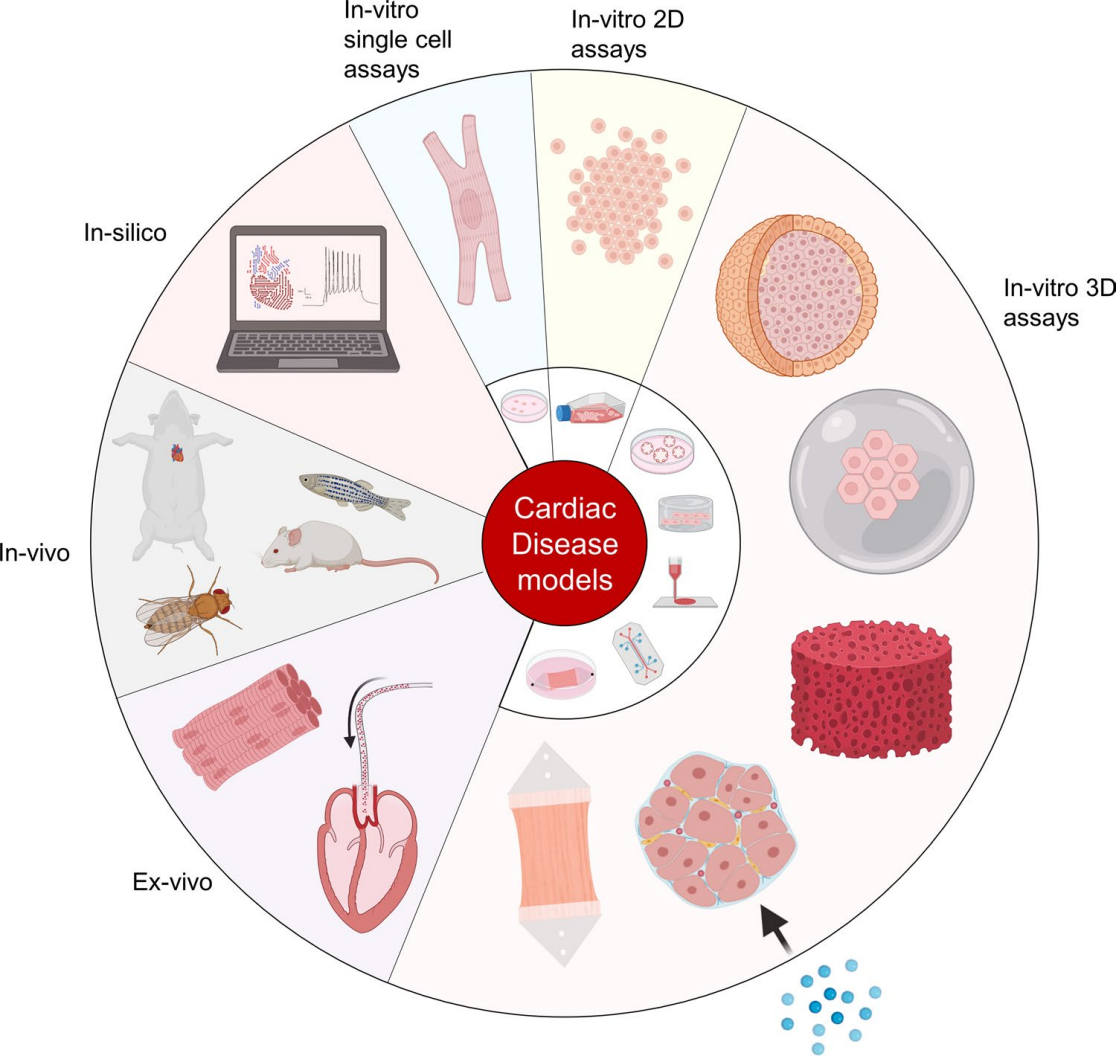
Overview of different cardiac disease models, including in vitro, ex vivo, in vivo, and in silico models. Image created in BioRender. 2D = two-dimensional, 3D = three-dimensional

Disease models are roughly divided into four separate categories, being in vitro models, ex vivo models, in vivo models and in silico models (Figure 1). Contemporary biomedical research is not equilibrated on the four categories presented here. The tendency leans towards in vitro model research as this field is currently most promising and innovative in providing more accurate cardiac model platforms. Yet, the whole spectrum will be discussed from simple in vitro platforms towards in vivo experimentation such as animal studies. Overall, inherent constrains of every model necessitate a clear research question in order to prevent overfitting of the model at hand.

## In Vitro Models

In vitro models are cell-based replicas of the human body, aimed at recapitulating smaller organ segments rather than reproducing whole living organisms. They were once introduced to fulfill a bipartite ambition: to better emulate human biology and to reduce the need for animal experimentation. In vitro studies permit detailed analyses while being convenient and simple. However, due to their general simplicity, in vitro models are less genuine reflections of the in vivo situation. In addition, more complex in vitro models complicate the deduction of cause-effect relationships, as outcomes may be the result of more than one element [7]. This resultant trade-off between simplicity and realism underlies the reason why in vitro model platforms frequently fail to achieve perfection in terms of disease modeling. In reference to serving as drug screening platforms specifically, it should be noted that in vitro constructs, including those mimicking cardiac disease, often lack vital facets of pharmacokinetics such as metabolite processing by the liver and kidneys, rendering them inapt to accurately evaluate drug efficacy, side effects, and toxicity ranks above tissue levels [8]. Consequently, research findings that have been established on in vitro platforms still need to be confirmed in more complex preclinical modalities, including animals and humans. In the context of cardiac disease modeling, in vitro models vary from single cell constructs towards near-physiological mimics of functional beating myocardium. Their reliability and translational capacity largely depend on their ability to resemble human biology.

## Single Cell Models

One may choose from several cardiac cell sources when building in vitro platforms that model cardiac disease [9–11], typically isolated cardiomyocytes (CMs) and stem cell-derived cells. Isolated CMs are animal or human cells, directly obtained from cardiac tissue specimen and subsequently cultured in vitro as single cell models or used for more complex modalities. Although the isolation and culture processes are rather simple, isolated CMs are terminally differentiated which prevents them from multiplying in vitro, limiting their availability. Human isolated cardiac cells presumably represent the best choice of cell origin but are scarce as they can only be yielded from small cardiac biopsies from cardiac surgery. This explains the contemporary use of mostly animal cells as isolated CM source, specifically neonatal rat cells, which naturally suffer from animal-human discrepancies. Pluripotent stem cells (PSCs), including embryonic stem cells (ESCs) and induced pluripotent stem cells (iPSCs), are undifferentiated cells which can undergo infinite self-renewal and engender derivates of all three developmental germ layers (ectoderm, mesoderm, and endoderm), including cardiac cells. ESCs are derived from the inner cells mass of blastocysts, firstly isolated from mouse embryos in 1981 by Evans and colleagues [12] and thereafter yielded from in vitro fertilized human embryos by Thomson et al. in 1998 [13]. The advent of iPSCs originated in the discovery that terminally differentiated cells including skin fibroblasts and blood cells can retrieve their pluripotent states by introduction of four key transcription factors [14, 15]. These iPSCs can subsequently be coaxed into differentiating into numerous cell types such as cardiac cells.

The infinite proliferative and differential potential of PSCs in addition to their human-rooted nature represent their biggest value in in vitro modeling. However, their use is typically challenged by suboptimal differentiation and achieving high levels of maturation. In addition, the use of human embryos still faces significant ethical concerns as the yield of blastocysts from the inner cell mass involves destruction of the embryo [16]. Addressing the current limitations regarding differentiation efficacy and maturation however render iPSCs the primary cell choice for cardiac platforms in the future.

In vitro, cardiac cells can be studied as single cell models or used for multi-cellular constructs. Both platforms are valuable and necessary to build a balanced and all-encompassing perspective on physiology and disease. Investigating cells on an individual level allows understanding of cellular behavior in absence of neighboring cells with which continuous crosstalk would otherwise occur [17]. This enhances insight into independent intracellular processes, such as mechanical properties [18–20], ion channel currents responsible for electrophysiological properties in patch clamp studies [21, 22], and expression profiles of CMs when studying cardiac disease [23–25] or regeneration [26, 27]. Challenges for single cell models reside in the difficulty of avoiding damage during isolation and culturing cells independently as many cells including CMs are usually thriving in a multicellular setting. The isolated environment therefore elicit adaptive remodeling, resulting in deviance from their the natural in vivo state [28]. Hence, new techniques are still being explored to optimize single cell modelling and analysis today.

## 2D Models

Conventional 2D cell culture has been in use since the early 1900s and contemporarily still constitutes the model of choice for the majority of in vitro research incentives [29]. 2D models rely on cell adhesion to a flat surface such as a coated petri dish, flask or well plate that provides the cellular monolayer with mechanical support. Their simplicity, accessibility and cost-efficiency render them attractive for many researchers. In 2D monolayers, cells have equal access to nutrients and growth factors resulting in homogenous cell growth and proliferation [30]. This increases the model’s reproducibility and thus its suitability for high throughput applications [31]. Cells are easily harvested from monolayers and many biomedical assays are available for 2D cell cultures at present. In addition, 2D cultures techniques have been improved and standardized for numerous cell types as opposed to more complex 3D modalities [30]. Yet simple and convenient, 2D models are also tied to inherent flaws. Mammalian hearts are composed of highly differentiated cells in a complex 3D arrangement that are mechanically, electrically, and biochemically coupled. A 2D monolayer that habitually consists of only one cell type cannot provide key cues that are essential for in vivo-like tissue building and cellular maturation.

Research has shown that cellular gene and protein expression changes when cells are cultured in 2D environments [30]. The lack of an intricately organized extracellular matrix (ECM) for the cells to adhere to undermines important cell-cell and cell-ECM interactions [32]. Additionally, a tissue constructed in vitro will never hold the mechanical, electrical, and biochemical features as in vivo without a well-mimicked ECM. Incorporating dynamical factors, simulating interactions with other organ systems, and integrating external influences are particularly challenging tasks for 2D models. Therefore, traditional 2D in vitro systems, although informative, inevitably deviate the in vivo state and prove unfit for queries that demand more accurate modeling.

Various approaches currently exist to improve 2D environments in vitro [30]. Firstly, iPSC-CMs that are passaged on Matrigel and subsequently overlayered with a second Matrigel layer have demonstrated to yield higher CM purities up to 98%, a technique referred to as the sandwich method [33]. Secondly, surface topography improves cell-cell interactions, enhances contractile force and better organization of sarcomeres as well as more in vivo-like cell morphology, attachment and spreading [34]. Lastly, the stiffness or Young’s modulus of the substrate that the cells adhere to significantly affects bioactivity. Muscle cells prosper on moderate substrate stiffnesses with Young’s moduli at roughly 8 to 17 kPa [35]. Utilizing these substrate stiffnesses can thus substantially enhance cellular purity, maturity, and behavior, and be easily applied to 2D systems.

Despite the challenge of 2D models to faithfully mirror in vivo habitats and human disease phenotypes, they remain indispensable to preclinical cardiac research. Importantly, 2D models occasionally prove to be better fits to certain research aims than other, more complex modalities. The platform of choice is indeed dictated by the specific process of interest for which 2D approaches may be superior due to their simplicity, accessibility for assays and/or the asset of well recapitulating tissue interfaces.

## 3D Models

Recent research has shifted towards the use of more complex 3D platforms aimed at more accurate modeling in in vitro settings. All 3D models are based upon three main components: (1) cardiac cells, either isolated or differentiated from PSC sources, (2) a 3D substrate or scaffold that will serve as ECM for cells to adhere to and (3) a chamber that provides an aseptic environment for cell culture and that allows for precise control of culture conditions, commonly referred to as the bioreactor [36]. Bioreactors can meticulously regulate oxygen levels, media fluid flow as well as integrate mechanical and electrical cues for better mimicry of in vivo conditions. For cardiac tissues specifically, incorporating cyclic mechanical strains, fluid shear stress from laminar blood flow, electrochemical gradients as well as proper biochemical cues is most vital to achieve resemblance.

3D models are chiefly being categorized according to scaffold type. These model types, although inherently different, share a common set of perks and drawbacks compared to 2D constructs. Firstly, 3D platforms represent better facsimiles of human physiology and disease as the 3D architecture in which cells reside enhances cell-cell as well as cell-matrix interactions, cell adhesion to ECM structures, cell migration and spreading throughout the matrix, cellular proliferation, growth, polarization, and morphology as well as maturation and differentiation in case of stem-cell derived cells. Secondly, due to the increased presence of cellular interplay, multicellularity is better achieved in 3D constructs using direct or indirect co-cultures [9]. Thirdly, 3D models allow for easier and more accurate disease induction as micro-environments and dynamical cues can be meticulously tuned with bioreactors. Fourthly, 3D constructs naturally engender gradients of oxygen and media throughout the tissue culture as opposed to monolayers in which cells are in direct exchange with nutrients to which they all have equal access to. In addition, monolayers are equally and directly exposed to drugs prompting the illusion of heightened drug efficacy. Lastly, cellular survival and maintenance of cellular quality is superior in 3D tissue cultures enabling the longer-term use of these models.

Drawbacks associated with 3D platforms include the common necessity of vast amounts of cells, approaching roughly 0.5 to 2 million cells per construct [36], rendering them more expensive and time-consuming. Additionally, no universal 3D system or protocol for fabrication currently exists as 3D models are often still exploratory and remote from standardization, presently complicating their use on larger scales and direct comparison in scientific literature. Moreover, cells and engineered tissue specimen are rather difficult to harvest once they have been incorporated into 3D constructs, which occasionally reduces their accessibility to assays. Challenges that remain for 3D platforms include the mimicry of whole heart remodeling, inter-organ interplay, influence of external factors and true reflections of multicellularity, vasculature networks, nerve meshes and immune system interactions.

### Hydrogel Model

Hydrogel models pioneered in the field of tissue engineering as the first-ever modality to model disease using 3D frameworks. It is still the most used technology for constructing 3D platforms [31, 37]. Hydrogels are gel-like structures composed of hydrophilic polymers that have either natural (gelatin, collagen, fibrin, alginate) or synthetic (polyglycolic acid (PGA), poly-e-caprolactone, polylactic acid (PLA)) origins [38]. They are initially liquid in form, mixed with cells and consequently crosslinked to a gel that entraps the cells in 3D shapes, provided by a casting mold. Hydrogels based on natural polymers gained much popularity as their molecular make-up matches that of in vivo ECM. In addition, they are biocompatible and biodegradable to similar extents as natural tissues and their mechanical, electrical, and chemical properties can be easily tuned. Hence, hydrogels are excellent materials to be used as scaffolds in engineered tissue constructs. However, the degradation velocity for hydrogels is rather high and therefore hard to match to the speed of new tissue formation to maintain tissue structure and integrity [30].

### Bioprint Model

3D printing techniques have flourished over the past few years and recently made their way towards applications in tissue engineering. Commonly, 3D objects of any shape are printed from digital models, using synthetic or natural materials such as hydrogels to serve as scaffolds for cell culture [9]. In some techniques, cells are seeded onto the scaffold after it has been printed as to construct a custom-shaped piece with cellular covering. However, cells are solely lining the surface rather than being merged within the matrix which results in poor scaffold cellularization and low cell penetration. The advent of bioprinting therefore proposed a more advanced alternative which involves using cell suspension as part of the ink, commonly referred to as bio-ink [9]. In addition, the approach is slowly paving the way for building personalized 3D cardiac models in which the 3D printed scaffold is based on CT or MRI images of the patient’s heart or vasculature system and in which cells are derived from the patient’s own reprogrammed stem cells.

### Decellularized Model

An alternative to the use of artificial matrices is employing the heart’s own ECM by decellularizing cadaveric animal or human hearts. Decellularization is aimed at removing the cellular constituent by perfusion, engendering a cell-free ECM scaffold that is completely natural and that contains acellular vascular conduits sufficient to support the heart [39]. Decellularized hearts are generally repopulated using animal-derived [40] or iPSC-derived cardiac cells [41], although issues of low cellularization efficiency exist. However, this technique comes with an inherent trade-off between preserving the natural ECM architecture and optimal cell removal as decellularization agents inevitably affect the matrix’s biochemical composition [39].

### Sandwich Model

The sandwich or cell sheet method involves layering several 2D monolayers, constructing a 3D model without the use of scaffolds [30]. Haraguchi and colleagues successfully fabricated a functional 3D system by stacking cell sheets of confluent cultured cells that pulsated spontaneously and synchronously [42]. The platform demonstrated that matrices are not an intrinsic necessity for developing well-beating cardiac patches. It does however evidently deviate the in vivo condition of cardiac tissue as lacking an accurately mimicked ECM will affect cellular maturation and in turn, morphology, and behavior. It does provide an easier, quick, and cost-effective method for building systems that suffice with lesser biological resemblance to the in vivo state.

### Spheroids and Organoids

Organoids are self-organized multicellular structures, derived from stem cells that can mimic the 3D architecture of in vivo tissues by self-assembly. They can be crafted to replicate much of the complexity of an organ and are frequently referred to as mini organs accordingly. The terms spheroids and organoids are often used interchangeably although they are characterized by distinctive traits. Organoids are complex constructs of organ-specific cells, derived from either normal or diseased stem cells that self-assemble when provided with a scaffolding extracellular environment. This offers the cells an ideal growth habitat with biological cues that aid in self-organization. Spheroids are simpler clusters of broad-ranging cells that do not require a scaffold or mechanical support as they simply aggregate into 3D structures within cell suspensions. Organoids typically exhibit polarity and cell migration whereas spheroids may or may not demonstrate polarity. All in all, organoids are more complex and in vivo-like, resembling the cell and tissue structures more precisely. This renders them fit for advanced disease and organ modeling, drug screening applications, and regenerative and personalized medicine. Spheroids are made with higher reproducibility rates and develop metabolic gradients which engenders more heterogeneous cell populations from the inside outwards. They can also well-recapitulate disease phenotypes and may be used on high throughput scales.

Cardiac organoids are optimally at least tricultured constructs that consist of CMs, cardiac fibroblasts (CFs) and endothelial cells (ECs). In this regard, CMs provide core beating function, CFs produce and maintain ECM and ECs regulate CM contraction and vasomotor tonus through autocrine as well as paracrine signaling in addition to transporting nutrients and waste [43]. Cardiac organoids can be made from iPSCs that are directed into atrial and ventricular cell types separately, which may then be combined to create an organoid that contains distinctive tissue regions as is true for in vivo myocardium [44]. Taking this a step further, cardiac organoids may be combined with other organoid-types such as lung organoids, kidney organoids and/or liver organoids in order to build a platform that allows for organ system interplay [45]. Lastly, organoids are also perfect model candidates for researching human embryonic development, organogenesis and tumorigenesis as their formation befalls without much of the researcher’s interference.

Even though organoid-based models do capture myocardial architecture better than most other 2D and even 3D platforms, they still deviate from in vivo human hearts. The drawbacks that PSCs are associated with automatically apply to organoids as well. Firstly, even multicellular organoids still lack the influence of immune cells, smooth muscle cells and nerve cells that play significant roles in vivo. Secondly, organoid-formation is a though process to precisely regulate and reproduce, resulting in individual models that differ in shape, size, architecture, and cellular composition. As previously stated, their genesis is chiefly endogenously driven with many molecular cues occurring beyond human control. Organoids may thus be deemed heterogeneous models, owing to individual diversity but also to the lack of a globally standardized protocol that causes outcome variations between research groups [46]. Thirdly, organoid formation generally requires millions of cells per model, which is a tenacious burden to overcome, bearing in mind the low efficiency of PSC-differentiation at present. Fourthly, the compact 3D structures of organoids tend to complicate advanced imaging due to limited imaging depth, the imaging dye diffusion barrier and light scattering properties that prevents staining the organoid core [43]. Fifthly, the uneven distribution of nutrients and oxygen throughout the organoid layers frequently results in necrotic cores which limits maximum model size. Most organoid models bestow on diffusion, but this could be improved by promoting organoid vascularization for which different approaches are being developed in recent years [47]. Finally, organoid models on itself still lack the pivotal effects of dynamical cues and external factors that are present in vivo. The latter may however be overcome by integrating organoid models into heart-on-a-chip platforms that are specialized in this field [45].

### Heart on-a-chip-models

Organ-on-a-chip models are microfluidic devices that culture living cells in continuously perfused chambers in order to simulate organ function. The concept does not intent to model whole organs but rather aims to build small units of cultured cells that replicate tissue function on microscopic levels. Microfluidic systems typically comprise of at least four elements: a microfluidic device consisting of chambers connected by microchannels, microtissue that is incorporated into the chambers of the system, a microreactor that holds the device and a set of microsensors for results readout.

Microfluidic systems provide oxygen and nutrient delivery via perfusion by linking cells in a fluidic manner using microchannels. The smallest microfluidic system or chip consists of only one chamber containing one or two kinds of cell type (e.g., CFs and/or CMs) that is perfused by only one microchannel. In more complex systems, multiple chambers perfused by two or more microchannels may be designed, each containing a variety of cultured cell types that are separated by semipermeable membranes from their microchannels to create in vivo like tissue- tissue interfaces and thus mimic physiological interactions [48]. As regards myocardium, atrial and ventricular tissue can be separately cultured on-a-chip to mimic the different chambers of the heart in vivo. The fact that culture medium flows continuously throughout the system rather than being static and replaced at set times points only is a major enhancement regarding biological mimicry compared to other modalities [49]. In addition, instead of using semi-static environments, microfluidic systems are able to independently integrate and regulate dynamic factors, such as physical forces, electrochemical gradients, biochemical stimuli, and fluid flow [50].

Disease induction is feasible through either genetic alteration of iPSC-derived cardiac cells or by simply modifying the features of the bioreactor and/or microfluidic design. Hypoxia-induced cardiac disease for example may be induced by modulating medium oxygenation, thereby mimicking temporary coronary occlusion [51]. Cardiac fibrosis induced by mechanical overload may be replicated through the exert of cyclic compression strains onto engineered cardiac tissue on-a-chip [52].

Furthermore, heart-on-a-chip models have shown to be more reproducible due to the requirement of precise and automatable manufacturing. This facilitates their use on larger scales for high throughput applications [5]. They also tend to be more cost-effective as a reduced number of cells, reagents and biomaterials are needed for microsystem construction. And by means of microsensors, microfluidic systems assist in monitoring cell status. This promotes assay-performance compared to other 3D modalities wherein cells are deeply embedded in 3D matrices [53].

Disadvantages of microfluidic systems are mainly tied to the challenge of obtaining mimicry of all myocardial features on the chip. Most heart-on-a-chip systems should be viewed as ventricle-on-a-chip models since the vast majority of current research uses ventricular cells only as this cardiac subtype can be cultured and differentiated with high purity. This is notwithstanding the undisputed relevance of other myocardial tissue components including the atria, conductive system and vasculature when modeling cardiac disease mechanisms, drug efficacy and toxicity [54]. Drug-related adverse conditions effecting the atrium such as atrial fibrillation elicit severe complications or even sudden death when administered to treat ventricular disease. Recapitulating the conductive system poses many challenges and even though we provide a microchannel-based platform that somewhat approaches a vascular-like network, there is much scope for improvement to accurately model this feature as it exists in vivo. Mimicking the concept of different heart chambers connected by blood vessels that are lined with endothelial cells becomes specifically relevant when utilizing the platform for drug screening. Conventionally, therapeutic agents are directly added to tissue culture media the engineered tissue is bathing in. In reality, drugs are delivered to cells through a vasculature system wherein endothelial cells meaningfully interact with administered drug agents. In addition to an often-fragmentary representation of the myocardium, heart-on-a-chip models usually lack inter-organ interplay and external factor influence although research is vastly expanding in this regard [45, 50, 55].

To date, various organs and tissues have been modeled on microfluidic platforms, which has prompted the opportunity of building a body-on-a-chip system containing multiple different organ-on-a-chip units as to simulate physiological organ interaction. Pires de Mello and colleagues developed a combined heart- and liver-on-a-chip system with a skin surrogate to evaluate drug toxicity of topically administered drugs [56]. Every organ and tissue type however still requires its own specific micro-environment, culture media and dynamical cues to grow into fully mature and in vivo-like cultures in vitro [50], which presently complicates their merge on microfluidic platforms.

Other drawbacks of organ-on-a-chip systems are the time-consuming nature and lack of global standardization. Advanced heart-on-a-chip models are often experimental, still uncovering their potential for improved (patho)physiological mimicry. Nonetheless, heart-on-a-chip models be deemed in vitro systems that currently best mimic the fundamental structural and functional units of native myocardium on microscale, providing a platform for accurate disease modeling as well as drug screening. Various heart-on-a-chip platforms have been designed and used at present to enhance our insight regarding (patho)physiological mechanisms that drive cardiovascular disease and uncover new potential therapeutic targets. Examples of these are models that mimic cardiac rhythm disorders [57], dilated cardiomyopathy [58], hypertrophic cardiomyopathy [59], hypoxia-induced myocardial injury [51], valvular disease [60], cardiomyopathy of Barth syndrome [61] and long-QT syndrome [62].

### Myocardial Slices

Myocardial slices are ultrathin (~300 μm) sections of living myocardium that have been presented as reliable in vitro modalities to address the issue of deficient in vivo mimicry [63]. The first-ever cardiac slices of ~ 400 μm were fabricated back in 1946 by Burdette and Wilhelmi [64] who studied the metabolism of rat hearts in response to hemorrhagic shock with high precision and reproducibility, using ventricles of rat hearts. Vitality and cardiac-like metabolism of these slices could be maintained up to 24 hours using oxygenated culture medium. The last thirty years have been dedicated to ameliorating fabrication and culture techniques including the application of near-physiological diastolic preload (1 mN) and continuous electrical stimulation in special cultivation chambers. This has facilitated the creation of (human) slices that currently demonstrate accurate in vivo structure and function for multiple weeks [65, 66]. Slice fabrication typically involves preparing living sections of the heart using a high-precision microtome, which usually leads to thin slices with only 2-3% CM damage in the superior and inferior cell layers.

The merit of the heart slice model lies in its 3D make-up but concomitant 2D-like simplicity. It therefore constitutes a model of intermediate complexity. The slices retain their native structure and function because they are directly produced from intact myocardium and their thinness ensures adequate oxygen and nutrient diffusion in vitro [63]. They do not require external perfusion like ex vivo modalities (e.g., myocardial wedges), which renders them particularly suitable for medium to high throughput systems as long as tissue availability is good. Additionally, myocardial slices may be produced from a range of animal models but more importantly, from human biopsies obtained from patients undergoing cardiac surgery which can reduce animal model usage and enhance clinical translation. Real-time assays are feasible within the platform due to high temporal and spatial resolution. Specifically relevant in the light of disease modeling is the option to produce heart slices from diseased individuals evading the absolute need of disease induction while facilitating personalized medicine. On the contrary, artificial disease induction may be established tuning the micro-environment of the bioreactor that holds the model [67]. Slices can be prepared from specific heart regions as desired by the researcher, including the ventricles, atria [68], endocardium, epicardium and with or without sections that carry the conductive system [69, 70]. In addition, they provide a means to explore regenerative applications [70] and more specifically, research on transplanted cardiac specimen [71, 72].

Drawbacks associated with myocardial slice models relate to the technical difficulty and skills required to produce high-quality slices, as well as the lack of current technique standardization which leads to notable variability between research groups. In addition, slice models may be surpassed by other in vitro models considering cost-effectiveness and tissue availability. The latter is tied to limited access to human heart specimen, complicating the use of human-based cardiac slice models on larger scales. Furthermore, when employing human-derived cardiac slices for disease modeling, control slices are usually taken from healthy unrelated individuals [73]. Non-matched samples will inevitably display outcome differences appertaining to inherent variations that are not related to the disease condition or interventions imposed. Additionally, slices are generated transmurally from endocardium to epicardium, enhancing the heterogeneity of cardiac samples. This should be accounted for when designing experiments as myocardial properties differ across the thickness of the heart muscle [67]. And although cardiac slices present great overall resemblance to in vivo myocardium, they are limited regarding the lack of capillary flow. Slices fully rely on diffusion, thus missing relevant endothelial cell interaction. Moreover, slices constitute an isolated myocardial unit, devoid of neuronal, hormonal as well as inflammatory impact or external influences. The heart slice model thus offers a valuable platform for researching isolated myocardial disease but lacks resources to examine complex heart disease or conditions that are significantly affected by either other organ systems or external cues. Nevertheless, slices do hold great promise in most faithfully mirroring human cardiophysiology and disease phenotypes, especially when incorporated into chip systems and multi-organ platforms.

### Ex Vivo Models

An ex vivo model refers to modeling of and experimentation on living tissues that are maintained in an artificial environment outside the body, by dint of perfusion and close mimicry of natural conditions, such as myocardial tissue wedges and ex vivo perfused hearts.

#### Myocardial Wedges

Tissue wedges are three-dimensional cardiac specimen, typically derived from the ventricle wall which offer advantages regarding in vivo-like 3D architecture, multicellularity, ECM resemblance and adult myocardial phenotypes. They fall short however regarding studying subacute and chronic disease conditions as they only remain viable for several hours. This also limits their suitability as drug screening platforms for long-term efficacy and toxicity evaluation. In addition, throughput for wedges is relatively low due to low sample yield per heart, the complexity of preparation and limited tissue availability. One heart can typically only provide one wedge and one or two papillary muscles [74].

#### Ex Vivo Heart Perfusion

Isolated whole heart perfusion was established by Elias Cyon in 1866 using frog hearts, which in time founded the base for the mammalian heart perfusion method in 1895 by Oscar Langendorff [75]. Langendorff mainly worked with extracted hearts from cats, dogs, and rabbits for which the overarching principle was to deliver blood through a cannula retrogradely in the ascending aorta, subsequently closing the aortic valve and forcing blood to enter the coronary arteries, hence facilitating myocardial perfusion [76]. Although the approach has been subject to numerous modifications over past years, current ex vivo heart perfusion models still display the pillars that were once shaped by the Langendorff principle or the more complex and later developed working heart method by Neely which employs ventricular filling via the left atrium and ejection via the aorta as occurs in vivo [75, 76].

The rat heart is by far the most popular animal species to be used for ex vivo preparations as it constitutes the perfect compromise between cardiac size, material costs, ease of handling and complexity of the experimental set-up [77]. In addition, they require lesser amounts of blood or perfusates and demonstrate smaller variability between individuals compared to larger animals, rendering them more affordable and reproducible candidates. Yet, larger animals present better mimicry to human hearts, but necessitate a blood-based or other oxygen-carrying perfusate, complicating the model use.

Ex vivo heart preparation typically exists of the following steps. First, the heart is excised under general anesthesia and immersed in cold perfusion solution (4 degrees Celsius) to reduce ischemic injury. Restoration of vascular perfusion should occur as soon as possible hereafter through aortic cannulation and coupling to a perfusion system. Once cannulation is completed and coronary perfusion is initiated, regular heart rhythm and contractile function will usually return within a few seconds [77]. When the heart is to be paced, electrodes may be placed within or onto the ventricle. Basic measurements include registration of heart frequency and contractile function, usually by means of an intraventricular balloon. In both methods, contractile function slowly deteriorates over time with a loss of 5-10% in cardiac output per hour. This may however be improved by periods of hypothermic arrests which has shown to preserve tissue function up to 24 hours or longer, the use of hemodialysis [78], or other methods that use blood conditioning such as plasma exchange [79–81].

The ex vivo heart model is particularly suited to study disease conditions involving ischemia and hypoxia, affecting the heart globally or regionally. Various grades of global ischemia can be incited through complete or partial clamping of perfusion lines or reducing fluid flow velocities [82]. Regional ischemia is usually instituted via ligation of specific coronary arteries after which reperfusion can be established by removing the ligature. The model provides a powerful tool for assessing these events in a continuous manner rather than sampling at set time points which would normally jeopardize the survival of an in vivo subject [77]. Additionally, the isolated heart serves as an ideal model for examining heart rhythm disorders [75] and arrhythmogenic effects of drug agents as well as for inventing strategies to improve ex vivo heart preservation [75, 83]. Lastly, cell-based therapy is gaining popularity in recent years, wherein cells are delivered into the heart to proliferate, differentiate into CMs, and induce angiogenesis within injured heart regions [75]. The end goal is to improve cardiac function, rendering proper assessment of these features vital. As isolated hearts are invaluable tools to evaluate cardiac functionality, several researchers have long been laying their focus on ex vivo heart preparations for studying such incentives [84, 85].

Definite perks associated with ex vivo heart perfusion concern good resemblance to in vivo heart anatomy, histology, and physiology as the platform is provided with an in vivo-like vasculature, furnishing fluid flow and shear stress, mechanical loading involving preload and afterload, electrical flow through the heart’s native conductive system and biochemical cues delivered by the perfusate. Importantly, research in this field occurs on organ levels rather than on tissue or cellular scopes. This renders simulation of inter-organ interplay more feasible compared to in vitro modalities as the perfusate can be finely tuned to mimic certain (patho)physiological conditions and deliver drug agents in in vivo-like manners. Dose-response studies may be well performed in perfused hearts with the option to perform washouts of administered drugs. Aside from examining hearts from healthy subjects, diseased hearts with known cardiac conditions could also be used for ex vivo heart preparations, possibly eliminating the need for artificial disease induction [75]. In comparison with in vivo models, it is also a relatively cheap, quick, and reproducible model to work with that offers great availability in case of animal-derived specimen, especially when working with smaller animal species [75].

Drawbacks concern the frequent need for interspecies extrapolation in case of animal-derived hearts as well as the ethical concerns that are tied to animal experimenting. A solution could be the use of slaughterhouse hearts, facilitating large amounts of hearts of animals sacrificed for food industry [78, 86]. The availability of human hearts is limited and although ex vivo perfusion of large animal hearts such as dogs, pigs and monkeys have been reported, they are less frequently used on account of logistic hindrances (high costs, need for large volumes of perfusion fluids etc.). Notably, isolated hearts undergo constant deterioration, limiting high-quality study of acute disease conditions to several hours. Moreover, control patients cannot be genetically matched to test subjects, leading to inevitable deviations between the test and control group results that do not derive from the disease condition or invention used. Ex vivo preparations are additionally limited due to the need of high coronary flow when using cell free perfusates to ensure sufficient oxygen delivery. This, in addition to the common insufficiency of oncotic proteins in the perfusate, renders the model prone to edema formation. Alternatively, blood-perfusion methods require large blood volumes, usually originating from a donor animal of a different species with the risk of contamination and immunogenic blood responses. This obstacle may be partly overcome by using crystalloid perfusion solutions supplemented with red blood cells [75]. However, the lack of blood-derived antioxidants and glucocorticosteroids would still render the preparation vulnerable to exogenous contamination, which would in turn elicit tissue responses that affect cardiac physiology. Other disadvantages include long learning curves and technical difficulties associated with model handling, the frequent occurrence of aortic valve insufficiency due to cannulation of the aortic root and retrograde perfusate flow, flow characteristics deviation of perfusate flow compared to in vivo coronary blood flow as well as the lack of the normal humoral background and extrinsic neuronal regulation of the heart. Lastly, the isolated perfused heart typically averts the presence of systemic disease conditions that commonly accompany cardiac disease, such as hypertension, diabetes mellitus and systemic atherosclerosis. These disease features affect the cardiac system to significant extents in vivo but are usually not simulated ex vivo [77]. Nevertheless, much of the limitations the model suffers from are outweighed by its benefits for specific research queries, including those broaching the field of cardiac physiology and pharmacology.

### In Vivo Models

#### Animal Models

Animal models have granted a significant contribution to our comprehension of the cardiac system. As of today, they still represent a cornerstone in preclinical research, including small animals (Drosophila, Zebrafish, Xenopus, mice, and rats), middle sized animals (Guinea pigs, rabbits, cats) and large animals (dogs, pigs, sheep, and non-human primates). However, criticism towards animal-based platforms is growing as inevitably, animal models represent imperfect facsimiles of human diseases and disorders with different genetic backgrounds and manners in which diseases develop [87]. Cardiac diseases are typically artificially induced in healthy animal subjects, an induction that occurs through either genetic, pharmacological, or surgical manipulation. Certain animal species prove better fits for certain induction methods. Mice have the ideal ability to make genetic models whereas surgical treatment is usually applied to larger rodents and larger animals such as rats, rabbits, and dogs [88]. Importantly, artificial disease induction never recapitulates the pathophysiological journey as it occurs in humans in vivo. It takes a short-cut and frequently, via another trigger than the original trigger in vivo. The goal of disease induction is to match the model phenotypically, a concept referred to as face validity. This dismisses the importance of the genetic and pathophysiological factors that created that disease, termed construct validity [4]. Suboptimal construct validity is most often present in animal disease models and has been a major bottleneck in the drug discovery process. In addition, research animals reside in specific, constant, and finely tuned laboratorial environments, devoid of any exposure to cues which human patients are dynamically exposed to in real life, such as microbes, weather changes, dietary varieties, and social events as well as variety in food, fluids, temperature, and humidity. Although this roots for controllability and reproducibility, it also renders true emulation of multifactorial disease more difficult. Compared to in vitro platforms, in vivo modalities are more time consuming, labor intensive and expensive but also variable and thus less reproducible. In addition, research intended on studying the long-term effects of disease or drugs is often challenging due to high costs and ethical objections, the latter of which mainly restricts the use of large animals that serve better models to study these events. Occasionally, subjective assessments of animal behaviors are necessary to investigate disease features or evaluate drug efficacy and toxicity such as pain, fear or discomfort, an aspect that relies on the interpretation of an observer rather than on objective measurements. Lastly, the majority of small animals including rodents have short life spans compared to humans. In these animals, the process of aging may be deemed relevant at 20 months and chronic disease may be redefined to weeks versus months or years in humans. Therefore, accumulation of age-related events as it occurs in humans may not be similarly present in smaller animal species. No ideal animal model exists that has proven superior for all cardiac research incentives. As a general principle, the larger the animal, the greater biological resemblance to humans with better translational applicability, but the more expensive, time consuming, labor intensive the research set-up becomes with logistical challenges being posed in addition to ethical issues being raised [4, 88]. Therefore, small animal models are generally deemed fit for proof of principle studies. Then, as insights expand, researchers convert to the application and validation of these concepts in larger animal models on smaller scales until translation to human clinical trials is deemed feasible.

Animal species can be matched rather meticulously depending on the cardiac process being studied according to the evolutionary correspondence to human counterparts. There are key publications [8, 87, 89–96] that intricately define these model types, and providing an extensive description is beyond the scope of this review. Nevertheless, it merits attention to underscore few of the most significant applications per species.

#### Small Animals

*Drosophila melanogaster*, commonly known as the fruit fly, occupies a vital niche in cardiac research owing to its genetic tractability and well-characterized genome. Researchers typically leverage Drosophila to explore questions related to cardiac morphogenesis, as their simple cardiac tube in fact displays developmental and functional resemblance to the vertebrate heart [89, 97, 98]. Furthermore, genetic studies occupy a prominent role in its application, as numerous genes, signaling pathways, and cellular processes involved in cardiac development exhibit a high degree of conservation and alignment with vertebrates, including humans [89, 99].

The transparent embryonic zebrafish (*Danio rerio*) additionally serves as a valuable asset, enabling real-time visualization of cardiac morphogenesis, encompassing the formation of the cardiac tube, chambers, and the establishment of the cardiac conduction system [100]. Notably, zebrafish possess a remarkable regenerative capacity for cardiac tissue following injury, a trait not shared by mammals [90]. Studying cardiac regeneration in zebrafish provides insights with potential implications for therapeutic strategies aimed at enhancing cardiac repair and regeneration in humans post-cardiac injury [101]. Additionally, zebrafish find applications in the investigation of congenital heart diseases through the induction of specific mutations, capitalizing on their genetic tractability [102]. They also facilitate efficient drug screening through high-throughput assays and support functional studies, thanks to their easily observable cardiac anatomy, enabling the comprehensive assessment of the effects of genetic mutations and drug treatments [90, 103].

*Xenopus laevis* (African clawed frog) and *Xenopus tropicalis* (Western clawed frog), are recognized for their large and robust embryos, which undergo external fertilization and ex utero development. Xenopus is a preferred animal model for the investigation of cardiac development and the associated molecular signaling pathways due to the ease with which gene expression can be manipulated in Xenopus embryos [104]. Similar to Drosophila and zebrafish, Xenopus embryos possess transparency, enabling real-time monitoring of cardiac function, encompassing parameters such as heart rate and contractility [105].

Transitioning to mammalian models, *Mus musculus* (mice) and *Rattus norvegicus* (rats) take central stages in animal modeling of cardiac disease, with both species offering unique advantages. Mice have genetic and physiological similarities to humans and genetically engineered murine models are considered instrumental to investigating specific cardiac genes and signaling pathways. For instance, researchers have developed mouse models to study the role of genes like MYH7 in cardiomyopathies [106] and genes related to ion channels in arrhythmias [107]. Rats, with their larger stature compared to mice, facilitate surgical interventions, enabling researchers to faithfully replicate human cardiac maladies such as ischemic heart disease and hypertension through techniques like coronary artery ligation [108, 109]. Their anatomical similarity allows for the investigation of surgical procedures and interventional therapies relevant to human patients.

#### Middle Sized Animals

Animals of intermediate size including cats, guinea pigs and rabbits are less commonly employed in animal experimentation; however, their utilization is species-dependent and characterized by distinct and specific applications. Cats, specifically domestic cats (*Felis catus*), have primarily been used in cardiac research to investigate cardiac conduction disorders, with a particular focus on ventricular arrhythmias [110]. This choice is attributed to the similarity in ventricular arrangement between feline and human hearts as well as the ease of induction of arrhythmias through methods such as electrical stimulation, surgical intervention or drug administration [111]. In a similar vein, the conduction system and electrophysiological characteristics of guinea pigs (*Cavia porcellus*) closely resemble those of humans. Consequently, they stand as optimal candidates for investigating cardiac electrophysiology and assessing the impact of anti-arrhythmic medications. Guinea pigs are preferred models for conducting studies related to prolonged QT intervals [112] and research on Torsades de Pointes [113].

Rabbits (*Oryctolagus cuniculus*) find specialized roles in research focused on atherosclerosis, lipoprotein metabolism, and the evaluation of how dietary factors and interventions influence cardiovascular health [114, 115]. This is primarily due to the heightened susceptibility of rabbits to diet-induced atherosclerosis, leading to the development of atherosclerotic plaques within arteries.

#### Large Animals

Canine models particularly permit the study of conductive physiology and rhythm disorders as well as studies based on heart rate, oxygen consumption and contractility due to key similarities with human physiology [95]. Specifically, they have served as suitable candidates for long QT syndrome (LQTS) research [116] and studies of Duchenne muscular hypertrophy [117], Brugada syndrome [118] and heart failure [119].

Porcine models (*Sus scrofa domesticus*) are distinguished for their anatomical congruity with the human coronary circulation [95, 120], which designates them as preeminent models for the investigation of myocardial ischemia and infarction [121]. Their utility extends to the examination of post-infarction remodeling [122], regenerative therapeutic modalities [123], and interventional cardiology procedures [124].

Ovine models (*Ovis aries*) chiefly play roles in the advancement of cardiac surgery, cardiovascular interventions, medical device testing, hemodynamic studies, pharmacological research, and cardiovascular imaging [125–127] due to their anatomical and physiological similarities to humans, combined with their manageable size.

Less frequently used models, including non-human primates such as macaques and baboons are frequently reserved for atherosclerosis research focused on the effects of dietary interventions [128], novel drug therapies and cardiac imaging studies [129].

Cardiac research endeavors often necessitate the use of diverse animal models, there is no single model that universally fits all research needs. It therefore seems fit to reiterate two questions before selecting an animal species for cardiac modeling: which (patho)physiological process is being studied and in what phase does the study reside (fundamental exploring versus more clinically invested research aimed at translation)?

Despite their shortcomings, animals do still play vital roles [87] in bridging the bench science to clinical practice for therapeutic drug development, clinical device testing and investigating complex disease biology as long as other preclinical modalities insufficiently grasp the intricacies of in vivo biological systems.

### In Silico Models

In silico or computational modeling represents an innovative addition to the myriad of preclinical research modalities and has been an expanding field in recent years. Experimental data is used to digitally recapitulate key features of the cardiac system in order to gain new insights into the complexity and dynamic interplays the heart is abundant in. Several scientists allege that in silico models hold promise for replacing the majority of in vitro and in vivo experimentation in the future as techniques gradually sophisticate [130].

The theory underlying computational modeling is complex and generally requires specific knowledge in digital data processing and analysis. Concisely, cardiac disease evolves through processes and cues that occur at different length scales as well as time scales. Referring to the first, the cardiac system could be layered in different orders of magnitude, from individual cells to the order of the pumping heart. Times scales refer to the system’s status and specifics at different points in time, during the heart’s pumping cycle. Computer models are able to concurrently integrate a multitude of different scales. This allows the model to evaluate the effects that changes in lower scales have on the system at higher scales, whereas in vitro modeling usually involves only one length scale and a set time frame at once. Generally, computer models can be classified into two categories, continuous and discrete models. Discrete models, also referred to as particle-based models, build up the subject particle by particle and thereby enable precise modeling on smaller scales with more detail, e.g., a specific heart region. Continuous models usually model on larger scales in which the cardiac system is composed of a multitude of different layers, with each layer owing its own specific features. These models simulate by way of using averages that can be applied to one whole model layer and are thus characterized by a lesser degree of meticulousness. Recently, hybrid approaches are being developed to model cardiac disease in attempt to merge the strength of both systems [130].

In silico models benefit from being generally human based and being able to grasp the intricacy of the cardiac system as well as to integrate (hemo)dynamic factors, interorgan system influences and environmental cues. In addition, they allow for studying the heart on different levels simultaneously, e.g., subcellular scales to individual levels [131] and tuning (patho)physiological circumstances to meticulous degrees. Logistically, computational modeling is cost-effective, quick, accessible, highly reproducible, non-invasive, and fit for many research queries.

Drawbacks tie to the ever-required input of data obtained from real-life experiments. The model only produces new data shaped and based on predictions using existing data and thus the model requires prescience on physiology, material characteristics, fluid dynamics and biochemistry, obtained from in vitro, ex vivo and in vivo modeling. Since knowledge acquired from in silico models is in essence assumption-based, it still needs to be validated in in vivo subjects.

In silico models have enabled research with four distinct objectives: modeling the impact of genetic mutations on cellular and organ function, establishing connections between molecular drug effects and resultant cellular and organ function, simulating cardiac pathology such as acute ischemia, and bridging the gap between molecular and preclinical physiological studies to clinical outcomes [132].

#### Single Cell Simulations

At the cellular level, computational models are harnessed for investigations into molecular mechanisms that lie at the base of cardiac pathology [132, 133]. In particular, action potentials of CMs may be simulated based on ion channel kinetics, calcium handling, and the interplay of subcellular components [134]. In recent years, mathematical models that encompass K^+^, Na^+^, and Ca^2+^ channels, in conjunction with various physiological processes like pH regulation and adrenergic stimulation have emerged. These models aim to forecast how mutations, medications, and physiological adjustments affect cellular and organ function. By example, Meier et al. [135] used highly detailed computational models to simulate the specific behaviour of Kv11.1-channel trafficking in CMs, shedding light on how mutations and temperature variations impact cellular function. Another illustration is provided by the investigation of cardiac arrhythmias, such as long QT syndrome, where in silico modeling allows simulation of ion channel dynamics in CMs affected by genetic mutations associated with this arrhythmia [136–138]. Lastly, the study of genetic mutations in silico is also applied for cardiomyopathies, including hypertrophic cardiomyopathy (HCM) [97], where computer simulations facilitate the analysis of how genetic mutations in myosin or troponin influence contractile function at the single cell level [139].

#### Multilayer Simulations

Single cell models can be advanced to larger structures, such as the myocardial wall and related tissue structures. This includes simulation of CM-CM coordination [140], electrical conduction [141], and contractile function [141] as well as cardiac substructures such as the atrioventricular (AV) node [142]. In research by Li et al., rabbit AV node tissue was stained to record electrical activity and visualize structural features. A precise 3D computer model of the AV node facilitated electrical behaviour simulations and was validated through experimental data comparisons, establishing its reliability for investigating cardiac dynamics. Another illustrative application for myocardial simulations is the study of myocardial infarction and post-infarction remodeling, in which in silico models replicate the spatial and temporal progression of ischemic damage to the heart [132, 143]. Computer simulations allow researchers to subsequently consider factors such as alterations in tissue geometry, fibrotic scar formation, and changes in electrical conductivity. In addition, computer models at the tissue level are instrumental in the examination of cardiac electrophysiology and arrhythmias. Researchers can simulate the propagation of electrical impulses through myocardial tissue and assess how structural abnormalities, such as fibrosis, affect conduction and the emergence of arrhythmias [138, 144]. These models help elucidate the arrhythmogenic substrates in conditions like atrial fibrillation [145] and ventricular tachycardia [146].

#### Whole Heart Simulations

While computational modeling of single-cells or specific cardiac regions and myocardial tissue structures offer valuable insights, significant potential of in silico models is harnessed through the orchestration of multi-scale simulations, founded on models at each of these hierarchies [132, 147]. This layered approach commences with the modeling of simple single-cell behaviours. Micro-level insights can then be seamlessly integrated into broader models, characterizing the behaviours of myocardial layers and substructures. These models, in turn, converge with those forecasting the dynamics of entire cardiac chambers, exemplified by the atria and ventricles [148]. The development of patient-specific electrophysiological heart models became possible through the availability of cardiac images from patients, usually MRI or CT, and was substantially propelled by the movement towards personalized medicine. In the study by Zheng et al. [149], a comprehensive whole-heart computer model was developed, using inputs based on patient data and realistic electrophysiological properties. This in silico model enabled the exploration of ventricular fibrillation (VF) mechanisms by incorporating factors like action potential duration restitution and conduction velocity restitution. It provided valuable insights into VF organization and the impact of electrophysiological variations on arrhythmia patterns. Gao et al. [150] built a computer model of the mitral valve (MV) and left ventricle (LV) using real-life patient data, including LV and MV geometry from cardiac magnetic resonance imaging (CMR). The model allowed simulations of cardiac function to explore MV-LV interactions and their relevance to cardiac disease. Additionally, in silico models are highly effective tools for simulating cardiac contractions during the cardiac cycle and comprehending the dynamics of blood flow within the heart chambers. These models faithfully replicate changes in myocardial tissue tension and deformation, providing valuable insights into the mechanics of effective cardiac pumping. Furthermore, they illuminate the interplay between hemodynamic processes, valvular function, and the interactions among blood and the structural components of the heart and conditions such as congestive heart failure [132, 146].

Ultimately, the power lies in blending interrelated submodels on different magnitude scales into one super simulation, like putting together a complex puzzle. This aids researchers to predict cardiac behaviour more accurately in response to an array of stimuli, including well-defined pathological triggers. For instance, it allows researchers to explore the cascade of pathological events, examining the molecular, cellular, and tissue-level responses involved, as well as the overarching pumping pattern of the entire heart [147, 151, 152]. Similarly, it affords the opportunity to scrutinize the consequences of a specific mutation, such as in a sarcomeric protein, and predict the effects of targeted interventions. However, it is crucial to emphasize that while these models offer great promise, their reliability and applicability hinge on the quality of the data and the accuracy of the underlying assumptions and mathematical representations.

In due course, as in silico models, including whole heart simulations, continue to evolve and align with the advancements in data quality and model accuracy, they are poised to provide invaluable insights into the complex world of cardiac dynamics and aid in the exploration of new therapeutic strategies.

## Summary

The scope of preclinical cardiac models is wide-ranging and characterized by a significant variety in model features and fields of application. This diversity underscores a pivotal attribute of preclinical research on a more general note: the perfect model does not exist, and a well-designed model or disease platform does not need to be optimally adequate for every research question in cardiac research. Every model furnishes approaches to a specific set of research aims, which may be either molecular-, organ- or individual-level. In addition, different models may be fit for examining distinct cardiac conditions, and for various fields of research, such as arrhythmias and heart failure or for examining disease etiology, developing diagnostic tools and novel therapeutic agents or the generation of prognostic data. This emphasizes yet another feature of preclinical research: it is inevitable to use multiple types of preclinical platforms in a step-like manner to acquire an optimal understanding of physiology and disease, prior to application in human clinical trials. These platforms range from cheaper and more accessible models that are used on large scales (high throughput) to more complex and expensive models that are used on smaller scales to confirm research findings in experimental set-ups with better biological mimicry. Another yet principal note concerns a model’s degree of complexity. Preclinical model systems vary greatly in this regard, in which in vitro models are deemed simpler retractions of biology compared to in vivo models, whereas ex vivo models occupy an intermediate level of complexity, bridging the gap between in vitro and in vivo. In turn, in vitro platforms and in silico models present their own array of complexity, due to differences in cell types used, scaffold choices, architecture degree of factor integration and the intricacy of the mathematical algorithms and the parameters used in the simulations respectively. The more complex a model system becomes, the more reliable and accurate it generally is, but the harder it will be to determine cause-effect relationships. Attaining maximum in vivo resemblance is therefore not always the goal when designing an experimental set-up. Alternatively phrased, a more complex model is not necessarily a better research model. It will remain the researcher’s responsibility to carefully investigate which model features are essential to answer their research aim and to outweigh their individual benefits and drawbacks (Table 1).

**Table 1:**
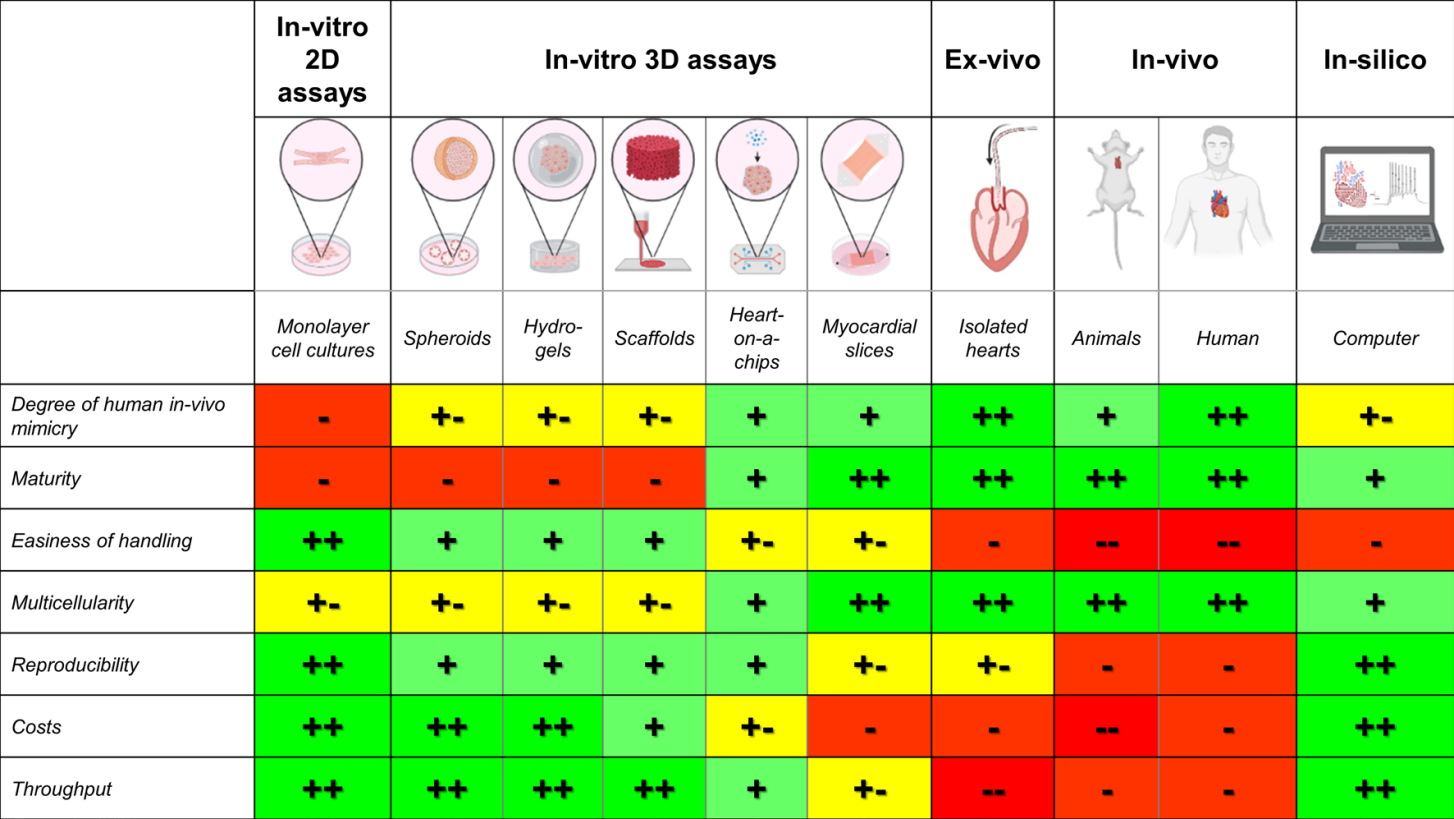
Overview of perks and drawbacks of different cardiac disease models. Image created in BioRender

2D = two-dimensional, 3D = three-dimensional

## References

[1] WHO. Cardiovascular Diseases (CVDs). Accessed 12 May 2022.

[2] Tsao CW (2023). Heart Disease and Stroke Statistics-2023 update: a report from the American Heart Association. Circulation.

[3] Denayer TST, van Roy M (2014). Animal models in translational medicine: validation and prediction. New Horizontal Transl Med.

[4] McGonigle P, Ruggeri B (2014). Animal models of human disease: challenges in enabling translation. Biochem Pharmacol.

[5] Annabi N (2013). Hydrogel-coated microfluidic channels for cardiomyocyte culture. Lab Chip.

[6] Ye L, Ni X, Zhao ZA, Lei W, Hu S (2018). The application of induced pluripotent stem cells in cardiac disease modeling and drug testing. J Cardiovasc Transl Res.

[7] Pitoulis FG (2022). Remodelling of adult cardiac tissue subjected to physiological and pathological mechanical load in vitro. Cardiovasc Res.

[8] Oh JG, Kho C, Hajjar RJ, Ishikawa K (2019). Experimental models of cardiac physiology and pathology. Heart Fail Rev.

[9] Lippi M, Stadiotti I, Pompilio G, Sommariva E (2020). Human cell modeling for cardiovascular diseases. Int J Mol Sci.

[10] Parrotta EI, Scalise S, Scaramuzzino L, Cuda G (2019). Stem cells: the game changers of human cardiac disease modelling and regenerative medicine. Int J Mol Sci.

[11] Moretti A (2010). Patient-specific induced pluripotent stem-cell models for long-QT syndrome. N Engl J Med.

[12] Evans MJ, Kaufman MH (1981). Establishment in culture of pluripotential cells from mouse embryos. Nature.

[13] Thomson JA (1998). Embryonic stem cell lines derived from human blastocysts. Science.

[14] Gurdon JB (1986). Nuclear transplantation in eggs and oocytes. J Cell Sci Suppl.

[15] Takahashi K, Yamanaka S (2006). Induction of pluripotent stem cells from mouse embryonic and adult fibroblast cultures by defined factors. Cell.

[16] Mlsna L (2011). Stem cell based treatments and novel considerations for conscience clause legislation. Indiana Health Law Rev.

[17] Yamada S, Nomura S (2020). Review of single-cell RNA sequencing in the heart. Int J Mol Sci.

[18] Ballan N, Shaheen N, Keller GM, Gepstein L (2020). Single-cell mechanical analysis of human pluripotent stem cell-derived cardiomyocytes for drug testing and pathophysiological studies. Stem Cell Rep.

[19] Nishimura S (2004). Single cell mechanics of rat cardiomyocytes under isometric, unloaded, and physiologically loaded conditions. Am J Physiol Heart Circ Physiol.

[20] Ribeiro AJ (2015). Contractility of single cardiomyocytes differentiated from pluripotent stem cells depends on physiological shape and substrate stiffness. Proc Natl Acad Sci USA.

[21] Verkerk AO (2021). Patch-clamp recordings of action potentials from human atrial myocytes: optimization through dynamic clamp. Front Pharmacol.

[22] Yamamoto Y, Hirose S, Wuriyanghai Y, Yoshinaga D, Makiyama T (2021). Electrophysiological analysis of hiPSC-derived cardiomyocytes using a patch-clamp technique. Methods Mol Biol.

[23] Miranda AMA (2022). Single-cell transcriptomics for the assessment of cardiac disease. Nat Rev Cardiol.

[24] Wang L, Hu S, Zhou B (2022). Deciphering cardiac biology and disease by single-cell transcriptomic profiling. Biomolecules.

[25] Gladka MM (2018). Single-cell sequencing of the healthy and diseased heart reveals cytoskeleton-associated protein 4 as a new modulator of fibroblasts activation. Circulation.

[26] See K (2017). Single cardiomyocyte nuclear transcriptomes reveal a lincRNA-regulated de-differentiation and cell cycle stress-response in vivo. Nat Commun.

[27] Honkoop H (2019). Single-cell analysis uncovers that metabolic reprogramming by ErbB2 signaling is essential for cardiomyocyte proliferation in the regenerating heart. Elife.

[28] Hodzic E (2016). Single-cell analysis: advances and future perspectives. Bosn J Basic Med Sci.

[29] Sacchetto C, Vitiello L, de Windt LJ, Rampazzo A, Calore M (2020). Modeling cardiovascular diseases with hiPSC-derived cardiomyocytes in 2D and 3D cultures. Int J Mol Sci.

[30] Duval K (2017). Modeling physiological events in 2D vs. 3D cell culture. Physiology (Bethesda).

[31] Weinberger F, Mannhardt I, Eschenhagen T (2017). Engineering cardiac muscle tissue: a maturating field of research. Circ Res.

[32] Stein JM, Mummery CL, Bellin M (2021). Engineered models of the human heart: directions and challenges. Stem Cell Reports.

[33] Zhang J (2012). Extracellular matrix promotes highly efficient cardiac differentiation of human pluripotent stem cells: the matrix sandwich method. Circ Res.

[34] Notbohm J (2019). Two-dimensional culture systems to enable mechanics-based assays for stem cell-derived cardiomyocytes. Exp Mech.

[35] Engler AJ, Sen S, Sweeney HL, Discher DE (2006). Matrix elasticity directs stem cell lineage specification. Cell.

[36] Savoji H (2019). Cardiovascular disease models: a game changing paradigm in drug discovery and screening. Biomaterials.

[37] Williams K (2021). A 3-D human model of complex cardiac arrhythmias. Acta Biomater.

[38] Zuppinger C (2016). 3D culture for cardiac cells. Biochim Biophys Acta.

[39] Tenreiro MF (2021). Interindividual heterogeneity affects the outcome of human cardiac tissue decellularization. Sci Rep.

[40] Robertson MJ, Dries-Devlin JL, Kren SM, Burchfield JS, Taylor DA (2014). Optimizing recellularization of whole decellularized heart extracellular matrix. PLoS One.

[41] Lu TY (2013). Repopulation of decellularized mouse heart with human induced pluripotent stem cell-derived cardiovascular progenitor cells. Nat Commun.

[42] Haraguchi Y (2012). Fabrication of functional three-dimensional tissues by stacking cell sheets in vitro. Nat Protoc.

[43] Nugraha B, Buono MF, von Boehmer L, Hoerstrup SP, Emmert MY (2019). Human cardiac organoids for disease modeling. Clin Pharmacol Ther.

[44] Zhao D, Lei W, Hu S (2021). Cardiac organoid - a promising perspective of preclinical model. Stem Cell Res Ther.

[45] Skardal A (2020). Drug compound screening in single and integrated multi-organoid body-on-a-chip systems. Biofabrication.

[46] Kim J, Koo BK, Knoblich JA (2020). Human organoids: model systems for human biology and medicine. Nat Rev Mol Cell Biol.

[47] Yu J (2021). Vascularized organoids: a more complete model. Int J Stem Cells.

[48] Bhatia SN, Ingber DE (2014). Microfluidic organs-on-chips. Nat Biotechnol.

[49] Cruz-Moreira D (2021). Assessing the influence of perfusion on cardiac microtissue maturation: a heart-on-chip platform embedding peristaltic pump capabilities. Biotechnol Bioeng.

[50] Kimura H, Sakai Y, Fujii T (2018). Organ/body-on-a-chip based on microfluidic technology for drug discovery. Drug Metab Pharmacokinet.

[51] Ren L (2013). Investigation of hypoxia-induced myocardial injury dynamics in a tissue interface mimicking microfluidic device. Anal Chem.

[52] Kong M (2019). Cardiac fibrotic remodeling on a Chip with dynamic mechanical stimulation. Adv Healthc Mater.

[53] Yang Q, Xiao Z, Lv X, Zhang T, Liu H (2021). Fabrication and biomedical applications of heart-on-a-chip. Int J Bioprint.

[54] Zhao H (2014). Microengineered in vitro model of cardiac fibrosis through modulating myofibroblast mechanotransduction. Biofabrication.

[55] Li ZA, Tuan RS (2022). Towards establishing human body-on-a-chip systems. Stem Cell Res Ther.

[56] Pires de Mello CP (2020). Microphysiological heart-liver body-on-a-chip system with a skin mimic for evaluating topical drug delivery. Lab Chip.

[57] Davis RP (2012). Cardiomyocytes derived from pluripotent stem cells recapitulate electrophysiological characteristics of an overlap syndrome of cardiac sodium channel disease. Circulation.

[58] Sun N (2012). Patient-specific induced pluripotent stem cells as a model for familial dilated cardiomyopathy. Sci Transl Med.

[59] McCain ML, Sheehy SP, Grosberg A, Goss JA, Parker KK (2013). Recapitulating maladaptive, multiscale remodeling of failing myocardium on a chip. Proc Natl Acad Sci USA.

[60] Chen MB, Srigunapalan S, Wheeler AR, Simmons CA (2013). A 3D microfluidic platform incorporating methacrylated gelatin hydrogels to study physiological cardiovascular cell-cell interactions. Lab Chip.

[61] Wang G (2014). Modeling the mitochondrial cardiomyopathy of Barth syndrome with induced pluripotent stem cell and heart-on-chip technologies. Nat Med.

[62] Itzhaki I (2012). Modeling of catecholaminergic polymorphic ventricular tachycardia with patient-specific human-induced pluripotent stem cells. J Am Coll Cardiol.

[63] Watson SA, Terracciano CM, Perbellini F (2019). Myocardial slices: an intermediate complexity platform for translational cardiovascular research. Cardiovasc Drugs Ther.

[64] Burdette WJ, Wilhelmi AE (1946). Respiration of heart muscle slices from rats in the terminal stage of hemorrhagic shock. Proc Soc Exp Biol Med.

[65] Fischer C (2019). Long-term functional and structural preservation of precision-cut human myocardium under continuous electromechanical stimulation in vitro. Nat Commun.

[66] Watson SA (2019). Biomimetic electromechanical stimulation to maintain adult myocardial slices in vitro. Nat Commun.

[67] Pitoulis FG, Watson SA, Perbellini F, Terracciano CM (2020). Myocardial slices come to age: an intermediate complexity in vitro cardiac model for translational research. Cardiovasc Res.

[68] Amesz JHG, Rooij M, Tiggeloven VP, Bogers AJ, Taverne Y (2022). Biomimetic cultivation of atrial tissue slices: a novel platform for in-vitro atrial arrhythmia studies. Sci Rep.

[69] Klumm MJ, Heim C, Fiegle DJ, Weyand M, Volk T, Seidel T (2022). Long-term cultivation of human atrial myocardium. Front Physiol.

[70] Maselli D, Matos RS, Johnson RD, Chiappini C, Camelliti P, Campagnolo P (2022). Epicardial slices: an innovative 3D organotypic model to study epicardial cell physiology and activation. NPJ Regen Med.

[71] Qiao Y (2019). Multiparametric slice culture platform for the investigation of human cardiac tissue physiology. Prog Biophys Mol Biol.

[72] Amesz JH, Langmuur SJJ, van Schie MS, Taverne Y (2022). Production of living myocardial slices from circulatory death hearts after ex vivo heart perfusion. JTCVS Tech.

[73] Thomas RC (2016). A myocardial slice culture model reveals alpha-1a-adrenergic receptor signaling in the human heart. JACC Basic Transl Sci.

[74] Meki MH, Miller JM, Mohamed TMA (2021). Heart slices to model cardiac physiology. Front Pharmacol.

[75] Skrzypiec-Spring M, Grotthus B, Szelag A, Schulz R (2007). Isolated heart perfusion according to langendorff–-still viable in the new millennium. J Pharmacol Toxicol Methods.

[76] Langendorff O (1895). Untersuchungen am überlebenden säugethierherzen. Arch Gesamte Physiol Mensch Tiere.

[77] Sutherland FJ, Hearse DJ (2000). The isolated blood and perfusion fluid perfused heart. Pharmacol Res.

[78] Kappler B (2019). Investigating the physiology of normothermic ex vivo heart perfusion in an isolated slaughterhouse porcine model used for device testing and training. BMC Cardiovasc Disord.

[79] McLeod JS (2020). Ex vivo heart perfusion for 72 hours using plasma cross circulation. Asaio J.

[80] Church JT (2017). Normothermic ex vivo heart perfusion: effects of live animal blood and plasma cross circulation. Asaio J.

[81] Tchouta L (2022). Twenty-four-hour normothermic perfusion of isolated ex vivo hearts using plasma exchange. J Thorac Cardiovasc Surg.

[82] Martin TP, MacDonald EA, Elbassioni AAM, O’toole D, Zaeri AAI, Nicklin SA, Gray GA, Loughrey CM (2022). Preclinical models of myocardial infarction: from mechanism to translation. Br J Pharmacol.

[83] Pinezich M, Vunjak-Novakovic G (2019). Bioengineering approaches to organ preservation ex vivo. Exp Biol Med (Maywood).

[84] Suzuki K (2004). Targeted cell delivery into infarcted rat hearts by retrograde intracoronary infusion: distribution, dynamics, and influence on cardiac function. Circulation.

[85] Bishawi M (2019). A normothermic ex vivo organ perfusion delivery method for cardiac transplantation gene therapy. Sci Rep.

[86] Kappler B (2020). Attenuated cardiac function degradation in ex vivo pig hearts. Int J Artif Organs.

[87] van der Velden J (2022). Animal models and animal-free innovations for cardiovascular research: current status and routes to be explored. consensus document of the ESC working group on myocardial function and the ESC working group on cellular biology of the heart. Cardiovasc Res.

[88] Milani-Nejad N, Janssen PM (2014). Small and large animal models in cardiac contraction research: advantages and disadvantages. Pharmacol Ther.

[89] Souidi A, Jagla K (2021). Drosophila heart as a model for cardiac development and diseases. Cells.

[90] González-Rosa JM (2022). Zebrafish models of cardiac disease: from fortuitous mutants to precision medicine. Circ Res.

[91] Bowley G (2022). Zebrafish as a tractable model of human cardiovascular disease. Br J Pharmacol.

[92] Hoppler S, Conlon FL (2020). Xenopus: experimental access to cardiovascular development, regeneration discovery, and cardiovascular heart-defect modeling. Cold Spring Harb Perspect Biol.

[93] Zaragoza C (2011). Animal models of cardiovascular diseases. J Biomed Biotechnol.

[94] Hornyik T (2022). Transgenic rabbit models for cardiac disease research. Br J Pharmacol.

[95] Oh JG, Ishikawa K (2018). Experimental models of cardiovascular diseases: overview. Methods Mol Biol.

[96] Camacho P, Fan H, Liu Z, He JQ (2016). Large mammalian animal models of heart disease. J Cardiovasc Dev Dis.

[97] Duncker DJ, Bakkers J, Brundel BJ, Robbins J, Tardiff JC, Carrier L (2015). Animal and in silico models for the study of sarcomeric cardiomyopathies. Cardiovasc Res.

[98] Zhang D (2011). Effects of different small HSPB members on contractile dysfunction and structural changes in a *Drosophila melanogaster* model for atrial fibrillation. J Mol Cell Cardiol.

[99] Elis S (2018). Genes involved in *Drosophila melanogaster* ovarian function are highly conserved throughout evolution. Genome Biol Evol.

[100] McCann T, Shrestha R, Graham A, Bloomekatz J (2022). Using live imaging to examine early cardiac development in zebrafish. Methods Mol Biol.

[101] Hu B (2022). Origin and function of activated fibroblast states during zebrafish heart regeneration. Nat Genet.

[102] Ma J (2022). Functional screening of congenital heart disease risk loci identifies 5 genes essential for heart development in zebrafish. Cell Mol Life Sci.

[103] Lu X, Lu L, Gao L, Wang Y, Wang W (2021). Calycosin attenuates doxorubicin-induced cardiotoxicity via autophagy regulation in zebrafish models. Biomed Pharmacother.

[104] Tandon P, Conlon F, Furlow JD, Horb ME (2017). Expanding the genetic toolkit in xenopus: approaches and opportunities for human disease modeling. Dev Biol.

[105] Bartlett HL (2010). Echocardiographic assessment of cardiac morphology and function in xenopus. Comp Med.

[106] Xia Y (2022). Investigating the pathogenesis of MYH7 mutation Gly823Glu in familial hypertrophic cardiomyopathy using a mouse model. J Vis Exp.

[107] Cartwright JH, Aziz Q, Harmer SC, Thayyil S, Tinker A, Munroe PB (2020). Genetic variants in TRPM7 associated with unexplained stillbirth modify ion channel function. Hum Mol Genet.

[108] Evis MJ, Kane KA, Moore MR, Parratt JR (1985). The effects of chronic low lead treatment and hypertension on the severity of cardiac arrhythmias induced by coronary artery ligation in anesthetized rats. Toxicol Appl Pharmacol.

[109] Ku HC, Chien DK, Chao CL, Lee SY (2022). Left anterior descending coronary artery ligation for ischemia-reperfusion research: model improvement via technical modifications and quality control. J Vis Exp.

[110] Rials SJ (1995). Effect of left ventricular hypertrophy and its regression on ventricular electrophysiology and vulnerability to inducible arrhythmia in the feline heart. Circulation.

[111] Bernikova OG (2018). Repolarization in perfused myocardium predicts reperfusion ventricular tachyarrhythmias. J Electrocardiol.

[112] Nagasawa Y, Sakai K, Kurimoto R, Kawakami S, Aimoto M, Takahara A (2020). Sensitivity of inhalation anesthetics isoflurane and sevoflurane for the drug-induced QT-interval prolongation in Guinea pigs. J Pharmacol Sci.

[113] Guns PJ, Johnson DM, Van Op J, den Bosch E, Weltens, and J. Lissens (2012). The electro-mechanical window in anaesthetized Guinea pigs: a new marker in screening for torsade de pointes risk. Br J Pharmacol.

[114] Nie X (2016). PET/MRI of hypoxic atherosclerosis using 64Cu-ATSM in a rabbit model. J Nucl Med.

[115] Momtazi-Borojeni AA, Abdollahi E, Jaafari MR, Banach M, Watts GF, Sahebkar A (2022). Negatively-charged liposome nanoparticles can prevent dyslipidemia and atherosclerosis progression in the rabbit model. Curr Vasc Pharmacol.

[116] Ter Bekke RMA (2019). Proarrhythmic proclivity of left-stellate ganglion stimulation in a canine model of drug-induced long-QT syndrome type 1. Int J Cardiol.

[117] Amoasii L (2018). Gene editing restores dystrophin expression in a canine model of duchenne muscular dystrophy. Science.

[118] Nishida K (2004). Canine model of brugada syndrome using regional epicardial cooling of the right ventricular outflow tract. J Cardiovasc Electrophysiol.

[119] Gabisonia K (2022). Proteome dynamics and bioinformatics reveal major alterations in the turnover rate of functionally related cardiac and plasma proteins in a dog model of congestive heart failure. J Card Fail.

[120] Duncker DJ (2022). Integrated control of coronary blood flow in exercising swine by adenosine, nitric oxide, and K(ATP) channels. Am J Physiol Heart Circ Physiol.

[121] Monguió-Tortajada M (2022). Acellular cardiac scaffolds enriched with MSC-derived extracellular vesicles limit ventricular remodelling and exert local and systemic immunomodulation in a myocardial infarction porcine model. Theranostics.

[122] Ladage D (2013). Stimulating myocardial regeneration with periostin peptide in large mammals improves function post-myocardial infarction but increases myocardial fibrosis. PLoS ONE.

[123] Copeland KM (2022). Investigating the transient regenerative potential of cardiac muscle using a neonatal pig partial apical resection model. Bioengineering (Basel).

[124] Heidt T (2019). Real-time magnetic resonance imaging - guided coronary intervention in a porcine model. Sci Rep.

[125] Roubertie F (2016). Cardiac contractility modulation in a model of repaired tetralogy of fallot: a sheep model. Pediatr Cardiol.

[126] Carney JP (2022). New model for the assessment of transcatheter aortic valve replacement devices in sheep. J Invest Surg.

[127] Haouzi P, Chenuel B, Sonobe T (2015). High-dose hydroxocobalamin administered after H2S exposure counteracts sulfide-poisoning-induced cardiac depression in sheep. Clin Toxicol (Phila).

[128] Karere GM (2019). Diet-induced leukocyte telomere shortening in a baboon model for early stage atherosclerosis. Sci Rep.

[129] Bert AA (2013). Transesophageal echocardiography in healthy young adult male baboons (*Papio hamadryas anubis*): normal cardiac anatomy and function in subhuman primates compared to humans. Prog Pediatr Cardiol.

[130] Owen B, Bojdo N, Jivkov A, Keavney B, Revell A (2018). Structural modelling of the cardiovascular system. Biomech Model Mechanobiol.

[131] Colquitt RB, Colquhoun DA, Thiele RH (2011). In silico modelling of physiologic systems. Best Pract Res Clin Anaesthesiol.

[132] Niederer SA, Lumens J, Trayanova NA (2019). Computational models in cardiology. Nat Rev Cardiol.

[133] Sutanto H, Lyon A, Lumens J, Schotten U, Dobrev D, Heijman J (2020). Cardiomyocyte calcium handling in health and disease: insights from in vitro and in silico studies. Prog Biophys Mol Biol.

[134] Bondarenko VE, Szigeti GP, Bett GC, Kim SJ, Rasmusson RL (2004). Computer model of action potential of mouse ventricular myocytes. Am J Physiol Heart Circ Physiol.

[135] Meier S, Grundland A, Dobrev D, Volders PGA, Heijman J (2023). In silico analysis of the dynamic regulation of cardiac electrophysiology by K(v) 11.1 ion-channel trafficking. J Physiol.

[136] Veerman CC (2013). Slow delayed rectifier potassium current blockade contributes importantly to drug-induced long QT syndrome. Circ Arrhythm Electrophysiol.

[137] Abdelsayed M (2017). Differential calcium sensitivity in Na(V) 1.5 mixed syndrome mutants. J Physiol.

[138] Holzem KM, Madden EJ, Efimov IR (2014). Human cardiac systems electrophysiology and arrhythmogenesis: iteration of experiment and computation. Europace.

[139] Campbell SG, McCulloch AD (2011). Multi-scale computational models of familial hypertrophic cardiomyopathy: genotype to phenotype. J R Soc Interface.

[140] Weiss DL, Ifland M, Sachse FB, Seemann G, Dössel O (2009). Modeling of cardiac ischemia in human myocytes and tissue including spatiotemporal electrophysiological variations. Biomed Tech (Berl).

[141] Margara F (2021). In-silico human electro-mechanical ventricular modelling and simulation for drug-induced pro-arrhythmia and inotropic risk assessment. Prog Biophys Mol Biol.

[142] Li J (2008). Computer three-dimensional reconstruction of the atrioventricular node. Circ Res.

[143] Sáez P, Kuhl E (2016). Computational modeling of acute myocardial infarction. Comput Methods Biomech Biomed Engin.

[144] Boyle PM, Del Álamo JC, Akoum N (2021). Fibrosis, atrial fibrillation and stroke: clinical updates and emerging mechanistic models. Heart.

[145] Vigmond EJ, Ruckdeschel R, Trayanova N (2001). Reentry in a morphologically realistic atrial model. J Cardiovasc Electrophysiol.

[146] Trayanova NA (2012). Computational cardiology: how computer simulations could be used to develop new therapies and advance existing ones. Europace.

[147] Lopez-Perez A, Sebastian R, Ferrero JM (2015). Three-dimensional cardiac computational modelling: methods, features and applications. Biomed Eng Online.

[148] Whiting R, Sander E, Conway C, Vaughan TJ (2022). In silico modelling of aortic valve implants - predicting in vitro performance using finite element analysis. J Med Eng Technol.

[149] Zheng Y, Wei D, Zhu X, Chen W, Fukuda K, Shimokawa H (2015). Ventricular fibrillation mechanisms and cardiac restitutions: an investigation by simulation study on whole-heart model. Comput Biol Med.

[150] Gao H, Feng L, Qi N, Berry C, Griffith BE, Luo X (2017). A coupled mitral valve-left ventricle model with fluid-structure interaction. Med Eng Phys.

[151] Palau-Caballero G, Walmsley J, Van Empel V, Lumens J, Delhaas T (2017). Why septal motion is a marker of right ventricular failure in pulmonary arterial hypertension: mechanistic analysis using a computer model. Am J Physiol Heart Circ Physiol.

[152] Sáez P, Peña E, Tarbell JM, Martínez MA (2015). Computational model of collagen turnover in carotid arteries during hypertension. Int J Numer Method Biomed Eng.

